# Cerebral collateral circulation in experimental ischemic stroke

**DOI:** 10.1186/s13231-016-0015-0

**Published:** 2016-03-01

**Authors:** Elisa Cuccione, Giada Padovano, Alessandro Versace, Carlo Ferrarese, Simone Beretta

**Affiliations:** Laboratory of Experimental Stroke Research, School of Medicine, University of Milano Bicocca, Via Cadore 48, 20900 Monza, Italy; PhD Programme in Neuroscience, University of Milano Bicocca, Monza, Italy; Milan Center for Neuroscience (NeuroMi), Milan, Italy

**Keywords:** Ischemic stroke, Experimental stroke models, Cerebral collaterals, Ischemic penumbra, Infarct size variability, Collateral therapeutics

## Abstract

Cerebral collateral circulation is a subsidiary vascular network, which is dynamically recruited after arterial occlusion, and represents a powerful determinant of ischemic stroke outcome. Although several methods may be used for assessing cerebral collaterals in the acute phase of ischemic stroke in humans and rodents, they are generally underutilized. Experimental stroke models may play a unique role in understanding the adaptive response of cerebral collaterals during ischemia and their potential for therapeutic modulation. The systematic assessment of collateral perfusion in experimental stroke models may be used as a “stratification factor” in multiple regression analysis of neuroprotection studies, in order to control the within-group variability. Exploring the modulatory mechanisms of cerebral collaterals in stroke models may promote the translational development of therapeutic strategies for increasing collateral flow and directly compare them in term of efficacy, safety and feasibility. Collateral therapeutics may have a role in the hyperacute (even pre-hospital) phase of ischemic stroke, prior to recanalization therapies.

## Background

Cerebral collateral circulation is a subsidiary vascular network which is dynamically recruited after arterial occlusion and may provide residual blood flow to ischemic areas. Cerebral collateral flow during acute ischemic stroke is highly variable among different individuals and is emerging as a strong prognostic factor either in unselected stroke patients and in patients treated with intravenous rtPA or endovascular recanalization therapy [1]. Experimental stroke models could play a crucial role for a deeper understanding of the adaptive and modulatory mechanisms of cerebral collateral circulation. This may promote the translational development of a new stroke therapy, based on the therapeutic modulation of collateral flow in the hyperacute phase of ischemic stroke prior to recanalization therapies [2].

Here, we review the current methods for assessing cerebral collaterals during acute ischemic stroke and the most promising collateral therapeutic strategies, focusing on experimental stroke models.

## Cerebral collateral circulation in humans and rodents

Many similarities, with some notable differences, exist between humans and rodents in term of cerebral collateral circulation. The circle of Willis includes the anterior communicating artery in humans, while this vessel is totally absent in rodents, whose proximal segments of anterior cerebral arteries (ACA) converge to form one single median artery called Azigos ACA. In case of occlusion of cervical arteries, the circle of Willis represents a compensatory system to rapidly redistributing blood flow in both species. In rodents, the pterygopalatine artery originates from the proximal internal carotid artery (ICA) and provide extracranial collateral connections between external carotid artery and ICA via many arterial branches to facial, orbital and meningeal districts. In both humans and rodents, each cerebral artery provides blood flow to its vascular territory ramifying along the cortical surface to form a pial arteriolar network, creating anastomotic connections among different vascular territories, known as leptomeningeal anastomoses (LMAs). LMAs are mostly developed between cortical branches of middle cerebral artery (MCA) and ACA or posterior cerebral artery. In case of proximal occlusion of a cerebral artery, dynamic blood flow diversion through these anastomoses may provide residual (retrograde) blood flow to the cortical surface of the occluded artery territory, distally from the occlusion.

## Assessment of cerebral collateral flow in acute stroke patients

**Fig. 1:**
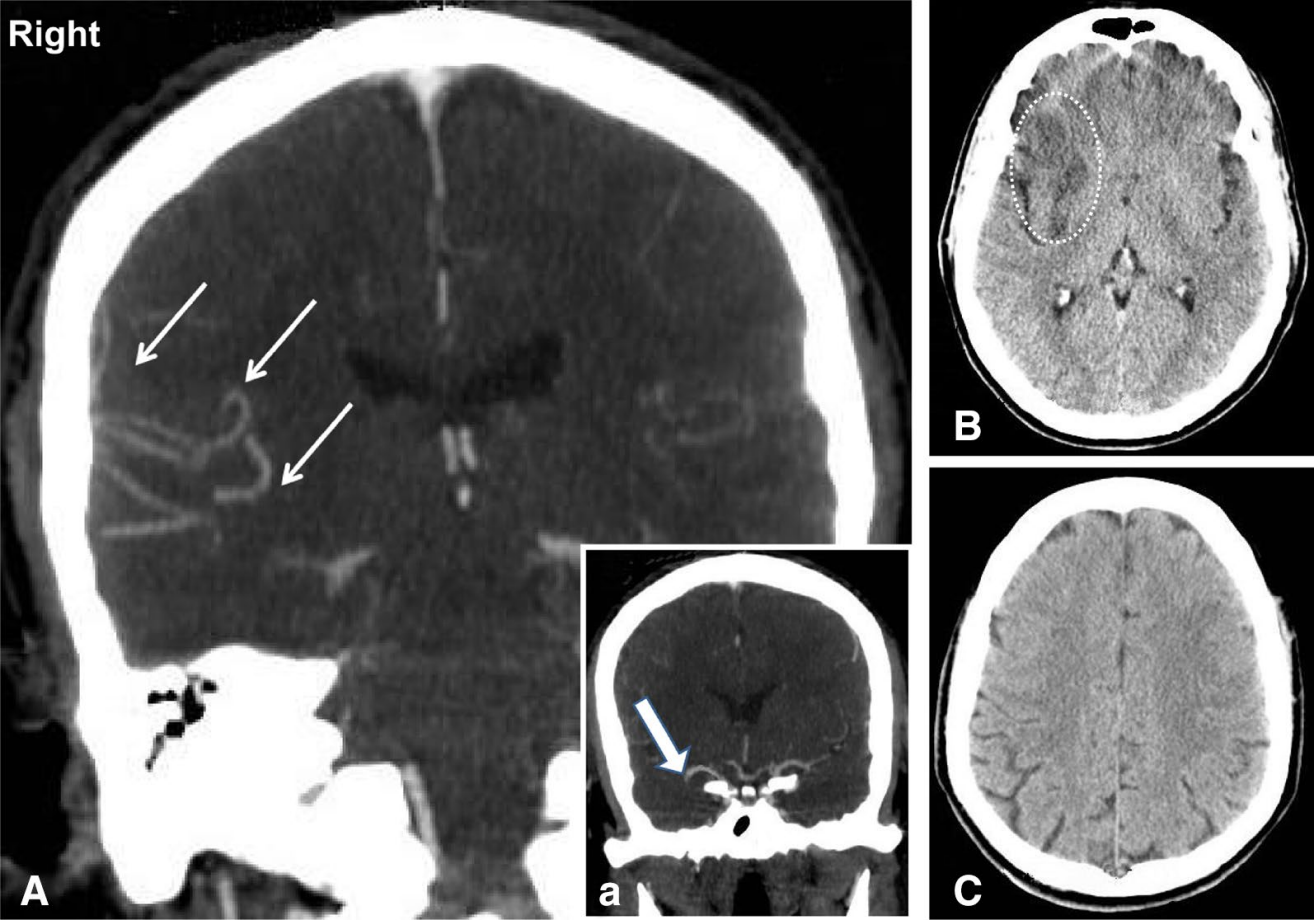
Clinical imaging of cerebral collaterals during acute ischemic stroke using CT-angiography. Collateral vessels [(**A**) *small arrows*] are visible in the right hemisphere. These vessels have been recruited after acute right MCA occlusion [(**a**) *large arrows*]. This patient was treated with intravenous thrombolysis and developed a small subcortical lesion (**B**), while the entire cortical territory was intact (**C**)

The anatomy of cerebral collaterals in acute stroke patients can be assessed using conventional digital subtraction angiography (DSA), CT angiography (CTA) or MR angiography (MRA), while their functional performance can be studied through tissue perfusion evaluation, via CT and MR perfusion techniques (PCT and PWI). At present, there is no agreement in clinical practice on which imaging should be performed, when after stroke and which patients would benefit most from cerebral collateral imaging. DSA is the gold standard for evaluating the recruitment of cerebral collaterals, but it is invasive and usually reserved for patients selected for endovascular procedures. CTA is able to provide direct visualization of collateral flow after arterial occlusion (Fig. 1) [3]. However, if imaging acquisition is done before the contrast arrives in the leptomeningeal vessels, there is a risk to underestimate the real extent of collaterals. Recently, multiphase CTA techniques have been developed to address this issue [4]. PCT allows to study the performance of collateral flow, which is indicated by preserved or increased cerebral blood volume (CBF) and augmented mean transit time [5]. Multimodal MRI provides a number of tools to assess collateral flow, although with some limitations. MRA can determine alterations of cerebral circulation within large cerebral arteries, with less spatial resolution compared to CTA [6]. FLAIR images on MR are able to show vascular hyperintensities distal to an occluded cerebral artery, due to the presence of a slow, retrograde blood flow in collateral vessels [7]. PWI could assess the performance of collateral flow, showing cerebral tissue with relatively preserved CBF and prolonged blood transit time [8]. Arterial spin-labelling MRI can detect brain regional hypoperfusion [9] and potentially identify the presence of leptomeningeal collateral routes [10].

## Assessment of cerebral collateral flow in experimental stroke models

In experimental stroke models, both the site and the duration of arterial occlusion are controlled. Continuous or repeated assessment of cerebral collateral flow could be performed, including pre-stroke assessment. For these reasons, preclinical research could play a crucial role for a deeper understanding of collateral response during cerebral ischemia and promote the translational development of collateral-based therapies. However, both cerebrovascular differences between different species and strains and inter-individual variability need to be meticulously considered to achieve effective results in this field [11].

Although some techniques used in stroke patients, such as DSA or MRI, could be used in stroke models for assessing cerebral collateral flow [12–14], significant limitations including costs, logistics and low spatial resolution prevent their widespread use. An easier assessment of collateral blood flow with great spatial and temporal resolution can be achieved using optical imaging and perfusion monitoring in experimental stroke models [15].

Laser speckle contrast imaging (LSCI) [16] provides maps of cortical blood flow, derived from the blurring of the speckle contrast pattern of a coherent light (laser), which is scattered by the motion of red blood cells (RBC) when directed to the cortical surface. Full-field imaging of the cortical surface and nearly real-time information about blood flow in both surface vessels and parenchyma are obtained. A cranial window is usually performed, although acquisition through intact skull is theoretically possible in mice. LSCI was used in rodent models of MCA occlusion (MCAO) to study changes in regional cerebral blood flow (CBF) and the dynamic response of LMAs to the vascular occlusion. After thromboembolic MCAO, blood flow establishment through pial arteriolar anastomoses was immediately evident, suggesting a prompt pathophysiological recruitment of the collateral circulation, also persisting after 24 h [17]. In another study [18], LMAs immediately provided blood flow after permanent MCAO and were classified in persistent, impermanent and transient on the basis of their dynamic changes. Though the speckle contrast values are indicative of RBC motion, they are not directly related to their speed or flow, with the exact relationship still undefined [19]. For this reason, LSCI can be used to measure relative blood flow changes, rather than for its absolute quantification [20].

In contrast to LSCI, two photon laser scanning microscopy (TPLSM) is an optical technique providing quantitative measure of blood flow velocity and direction in single vessels, with depth resolution up to 1 mm. Single arterioles, venules and capillaries of both surface and subsurface vasculature are resolved after intravenous injection of dextran conjugated with a fluorescent dye. A cranial window is required and scanning procedure is time-consuming. Collateral response after occlusion of both pial and penetrating arterioles in rats [21] were studies using TPLSM.

**Fig. 2:**
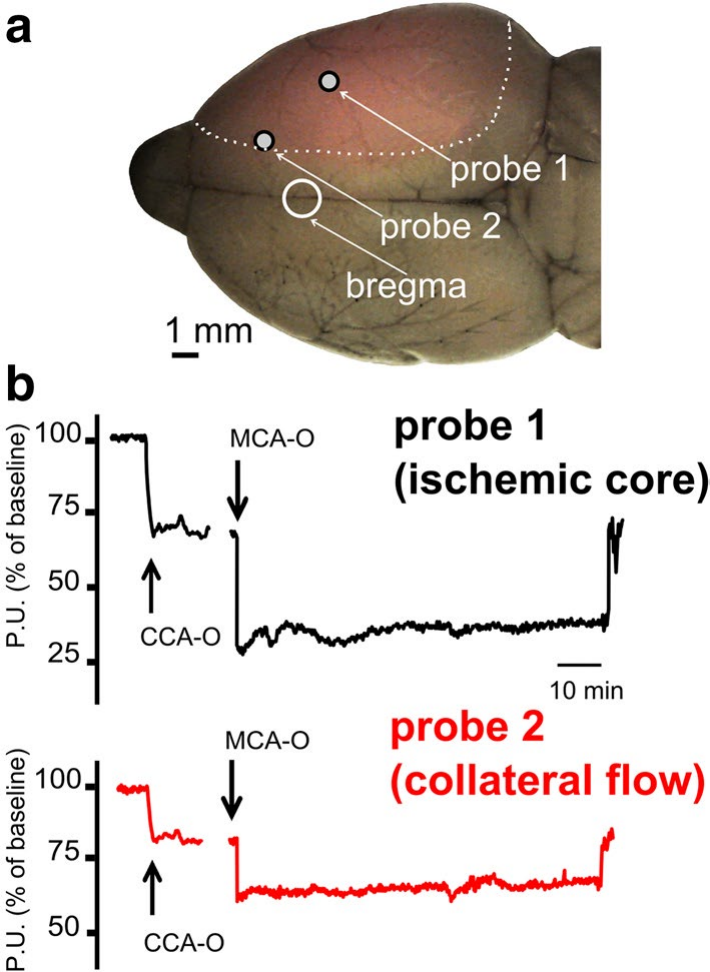
Monitoring of cerebral collateral flow in experimental ischemic stroke using multi-site Laser Doppler flowmetry. **a** The positions of the Laser Doppler probes are shown, with reference to their underlying MCA territory (*white dotted line*) and bregma. Probe 1 = central MCA territory (ischemic core; −1 mm from bregma, 5 mm from midline); Probe 2 = MCA–ACA borderzone territory (collateral flow; +2 mm from bregma, 2 mm from midline). **b** Laser Doppler tracings are shown from a representative animal showing a larger perfusion deficit in Probe 1 compared to Probe 2 during MCAO, suggesting functionally active intracranial collaterals under ischemic conditions. *P.U.* perfusion units

Laser-Doppler flowmetry (LDF) is a well-established technique for tissue perfusion monitoring and is recommended to confirm successful occlusion and exclude subarachnoid hemorrhage in experimental ischemic stroke [22]. Optical probes can be located on the cerebral cortex or skull, providing an integrated reading of the underlying pial vasculature and parenchymal capillary bed in 1 mm^3^ volume. Real-time relative cortical CBF values are obtained, while absolute CBF quantification cannot be achieved [23]. Our group developed an optimized system for multi-site LDF monitoring in rats during transient MCAO [24]. A custom made holder for two probes was attached to the intact skull to allow continuous monitoring of cerebral perfusion in the central MCA territory (Probe 1) and in the borderzone between ACA and MCA territories (Probe 2) (Fig. 2a). Although not providing a direct imaging of the LMAs, multi-site LDF monitoring allows real-time assessment of cerebral perfusion in two hemodynamically distinct territories during MCAO (Fig. 2b). Perfusion deficit recorded by Probe 2 is considered an index of the functional performance of LMAs, while perfusion deficit recorded by Probe 1 is used to confirm occlusion and reperfusion. Multi-site LDF may represent an easy method to quantify the functional activation of LMAs during ischemia in experimental stroke models and assess the effect of treatments.

The use of any of these methods (or a combination of them) to monitor arterial occlusion and collateral perfusion cannot be over emphasized to improve accuracy of pre-clinical stroke research. Advantages and drawbacks, in terms of temporal and spatial resolution, invasiveness and affordability of each technique are shown in Table 1.

**Table 1:** Methods for the assessment of collateral blood flow in experimental stroke models

Method	Temporal resolution	Spatial resolution	CBF information	Invasiveness	Cost
MRI	Not real-time	Whole brain with low resolution	Perfusion maps	None	High
LSCI	Almost real-time	Strictly surface reading	Relative CBF values	Craniotomy may be necessary	Moderate
TPLSM	Repetitive scanning required	Depth resolution	Quantitive CBF velocity and direction in single vessels	Craniotomy necessary	High
LDF (multi-site)	Real-time monitoring	Integrated reading in 1 mm^3^ cortical volume	Relative CBF values	Craniotomy not necessary	Moderate

## Cerebral collateral flow as stratification factor in neuroprotection studies

Despite over 1000 putative neuroprotective agents obtained promising results in experimental stroke models [25], no successful translation has occurred in the phase-3 stroke clinical trials performed so far [26]. Poor methodology of preclinical studies, including study design, heterogeneity of stroke models and stroke severity, time window, drug targeting, effective dose-finding and outcome assessment has been advocated as one of the main reasons of this failure in translation [27–29].

A well-recognized limitation of preclinical stroke models is outcome variability [11], particularly regarding infarct size [30] which is the most commonly used primary outcome. In a recent meta-analysis of 502 control groups in preclinical stroke experiments, the average infarct size coefficient of variation was about 30 % (ranging from 1.7 to 148 %) [31]. The problem with high outcome variability is that a higher number of animals is needed to get an adequate statistical power, which is problematic from both an ethical and economical point of view. The main reasons of infarct size variability in stroke modes are not completely understood. Although rat strain, surgical procedures, occluding filaments, anaesthesia and physiological monitoring have been demonstrated to be associated with infarct size variability [22, 32], factors related to inter-individual differences in cerebrovascular anatomy [33] and cerebral collateral circulation [34] have been reported. Interestingly, the National Centre for the Replacement, Refinement and Reduction of Animals in Research (NC3Rs) is currently funding a project (2014–2015) entitled “Determining the source of variability within experimental stroke models”, which is mostly focused on vascular anatomy and reperfusion [35].

**Fig. 3:**
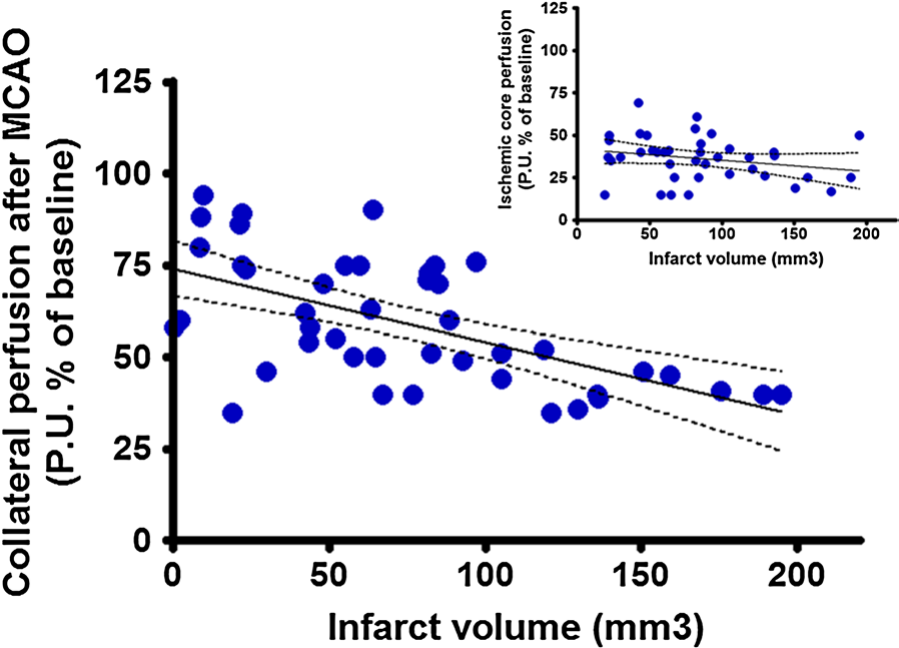
Relationship between cerebral collateral flow during MCAO and stroke outcome in rats. Linear regression between infarct volume and perfusion deficit during MCAO in the territory of leptomeningeal collaterals, measured using multi-site laser Doppler, was calculated for 45 consecutive untreated rats (p < 0.0001; Pearson’s r = −0.59). Notably, the correlation between infarct volume and perfusion deficit in the ischemic core (central MCA territory) was not significant (p = 0.14, smaller graph)

The variability of cerebral hemodynamics during ischemia has been largely neglected in preclinical research, as well as the influence of drugs on CBF [36]. Monitoring CBF, including cerebral collateral flow, may help to detect indirect neuroprotective effects in preclinical studies and predict outcome variability between treatment groups. Similarly to humans, the functional performance of collateral circulation during cerebral ischemia displays inter-individual variability in rodents [37]. Our group showed that the functional performance of the cerebral collaterals during MCAO in rats, assessed using multi-site LDF monitoring, predicted infarct size and functional outcome more accurately than conventional perfusion deficit in the ischemic core [34]. Further experiments using the same method, in a series of 45 untreated animals, confirmed a highly significant correlation of collateral flow during MCAO and stroke outcome (Fig. 3; unpublished results).

Animal stratification by collateral flow during MCAO represents a promising tool to adjust for outcome variability in experimental stroke studies. Using cerebral collateral flow during MCAO as a covariate in multiple regression analysis may represent a simple method to stratify animals in term of pre-treatment perfusion deficit, reducing the within group variability and improving efficacy analysis in preclinical neuroprotection studies. Further studies are needed to determine the more suitable method, timing and statistical tool for collateral flow assessment in pre-clinical neuroprotection trials.

## Acute therapeutic modulation of cerebral collateral flow

Intravenous thrombolysis with rtPA (Alteplase) within 4.5 h from symptom onset (for any vessel occlusion) and endovascular thrombectomy within 6 h from symptom onset (for large vessel occlusion) are currently the best therapeutic options for acute ischemic stroke [38, 39]. Unfortunately, recanalization is not always successful and, even when achieved, may be futile because of delayed reperfusion, hemorrhagic transformation, re-occlusion or vascular collapse downstream [40, 41]. Vascular aspects beyond the occlusion are often neglected [42]. Nonetheless, modulating collateral blood flow in order to augment or maintain perfusion to the ischemic penumbra could represent a new therapeutic strategy for the hyperacute (even pre-hospital) phase [2], particularly if applied before recanalization or neuroprotective therapies. Although different strategies (summarized in Table 2) could be used to modulate cerebral collateral flow during acute ischemic stroke, extensive research is needed in both animal models and stroke patients to establish the best approach in term of benefit-to-risk ratio.

**Table 2:** Potential strategies for modulation of cerebral collateral flow in acute ischemic stroke

Strategies		Risks	Cost	Results in preclinical stroke models	Results and feasibility in human stroke
Pressure load					
Induced hypertension		Haemorrhagic transformation, cardiac arrhythmias, myocardial ischemia	Low	Core and penumbra CBF augmentation through LMAs after distal MCAO in mice [43]	Preliminary results indicate efficacy (small clinical studies) [44, 45]. High feasibility
Intravascular volume load					
Dextran and hydroxyethyl starch		Anaphylaxis, pulmonary edema, platelet dysfunction	Low	CBF augmentation and improved outcome in various stroke models [46]	No benefit in early clinical trials (before the introduction of recanalization therapies) [47]. High feasibility
Albumin		Pulmonary edema, allergic reactions	Moderate	Cerebral perfusion enhancement through LMAs after distal MCAO in mice [48, 49]	No benefit in a large RCT (administered after recanalization therapy) [50]. High feasibility
Cerebral vasodilation					
Nitric oxide inhalation		Pulmonary irritation	Moderate	Selective arteriolar vasodilation in the penumbra and cortical CBF enhancement after MCAO in mice [52]	No results available in human stroke. Moderate feasibility (inhalation delivery equipment needed)
Sphenopalatine ganglion stimulation		Invasive (minor surgery)	High	Cortical arterioles vasodilation and CBF augmentation after photothrombosis [53]	Ongoing clinical trial [54]. Moderate feasibility (surgery needed)
Sensory-induced vasodilation		No risks known	Low	Gradual reperfusion through collaterals after MCAO in rats [56]	No results available in human stroke. High feasibility
Acetazolamide		Paraesthesia, nausea, metabolic acidosis	Low	Negative effect on outcome if administered 48-54 h after the onset of permanent MCAO [59]	No results available in human acute stroke. Clinically used as diagnostic tool in chronic stroke. High feasibility
Cerebral flow diversion					
Head down tilt		Increase in intracranial venous pressure	Low	Cerebral perfusion augmentation after bilateral CCAO in mice [62]	Increase in cerebral perfusion and blood flow velocity by flat head positioning (case series) [60, 61]. High feasibility
Partial aortic occlusion		Invasive (endovascular surgery)	High	Blood flow enhancement through LMAs after thromboembolic MCAO in rats [63]	Clinical trial suggest efficacy in post hoc subgroup analysis (further confirmation required) [64]. Moderate feasibility (endovascular procedure needed)

Increasing systemic blood pressure represents a first strategy. Phenylephrine, a selective α1-adrenergic receptor agonist, causes systemic vasoconstriction with very limited effects on cerebral vessels. A 30 % augmentation of blood pressure obtained through phenylephrine infusion after distal MCAO induction in mice enhanced cortical CBF both in core and penumbra [43]. In small clinical studies, norepinephrine- o phenylephrine-induced hypertension improved outcome in stroke patients [44, 45], but collateral circulation was not directly assessed, leaving its contribution unclear.

Increasing intravascular volume may represents a second strategy. Cerebral blood volume augmentation by plasma expansion and haemodiluition could improve cerebral perfusion in experimental stroke models [46]. However, in acute stroke trials performed in the 1990s, plasma expansion by dextran 40 and hydroxyethyl starch showed no benefit on neurological outcome or mortality [47]. Notably, all these early clinical studies were performed in the pre-thrombolysis era and outside a meaningful therapeutic window (patients were enrolled many hours or even days after symptom onset) and cerebral collateral flow was not assessed. Intravenous albumin administration has been reported to enhance cerebral perfusion and provide neuroprotection in preclinical works [48, 49]. However, a large randomized clinical trial showed no clinical benefit of intravenous albumin solution 25 % in ischemic stroke patients compared to standard treatment [50]. Notably, 85 % of these patients were treated with rtPA and intravenous albumin was administered on average 60 min after (not before) recanalization therapy.

Induction of selective cerebral vasodilation is a third strategy. Nitric oxide (NO) is a strong endogenous vasodilator with therapeutic potential for ischemic stroke [51]. NO inhalation following MCAO in adult mice induced a selective arteriolar vasodilation within the ischemic penumbra, likely through collateral arterioles, leading to decreased brain damage and improved functional outcome [52]. No results are available for inhaled nitric oxide in acute stroke patients. Sphenopalatine ganglion (SPG) electrostimulation activates parasympathetic fibers innervating intracranial vessels leading to their vasodilation. In preclinical studies, SPG-stimulation started after MCAO preserved DWI–PWI mismatch and reduced infarct size [53]. SPG electrostimulation in ischemic stroke patients has been demonstrated to be safe [54]. Stimulating cerebral function during ischemia could non-invasively enhance collateral perfusion of affected regions through neurovascular coupling mechanisms (i.e., functional hyperaemia) [55]. Sensory cortical activation induced by whiskers stimulation in rats lead to a gradual reperfusion via MCA distal collaterals, when the treatment was initiated within a critical time window from MCAO onset [56]. No results are available for sensory stimulation in acute stroke patients.

Selective cerebral arteriolar vasodilation could be obtained using acetazolamide, which inhibits carbonic anhydrase and consequently augments CO_2_ levels, causing pial arteriolar vasodilation and increased cortical perfusion in piglets [57]. In clinical practice, acetazolamide is used to test hemispheric cerebrovascular reactivity in patients with chronic cerebrovascular occlusions [58]. Quite surprisingly, the only report of acetazolamide in experimental stroke dates back to 1971 [59], was performed in cats undergoing permanent MCAO (without reperfusion) and the drug was administered using a very late time window (48–54 h after the onset ischemia). No results are available for acetazolamide in acute stroke patients.

Cerebral flow diversion is a fourth strategy. Gravitational influences of head positioning after acute vascular occlusion may affect pressure gradients in cerebral circulation, which enhancement may promote leptomeningeal recruitment. Augmentation of cerebral perfusion and increased MCA blood flow velocity has been reported in stroke patients after flat head positioning [60, 61] and after 5° head-down tilt following bilateral CCAO in mice [62]. A temporary partial occlusion of the abdominal aorta may divert flow from the splanchnic circulation. Transient aortic occlusion increased blood flow through ACA–MCA LMAs after thromboembolic MCAO in rats, restoring it to baseline levels and maintaining stroke-induced vasodilation [63]. A randomized clinical trial of this procedure in acute ischemic stroke patients demonstrated an acceptable safety profile and suggested efficacy in post hoc subgroup analysis [64].

## Conclusions

A limited number of clinical and preclinical stroke studies focused on cerebral collateral circulation. Generally, neuroprotective effects are being sought, whereas the contribution of collateral blood flow is rarely considered or just inferred. Preclinical stroke research has the potential to directly study the adaptive capacity and modulatory mechanisms of cerebral collateral flow during focal cerebral ischemia, using different methods and in different experimental conditions. These preclinical efforts are likely to be worthwhile and may produce useful translational concepts and direct comparisons of the different strategies to enhance cerebral collateral flow, including some therapeutic approaches which did not prove successful in past clinical trials conducted in the pre-thrombolysis era.
